# GABA_A_ receptors in visual and auditory cortex and neural activity changes during basic visual stimulation

**DOI:** 10.3389/fnhum.2012.00337

**Published:** 2012-12-31

**Authors:** Pengmin Qin, Niall W. Duncan, Christine Wiebking, Paul Gravel, Oliver Lyttelton, Dave J. Hayes, Jeroen Verhaeghe, Alexey Kostikov, Ralf Schirrmacher, Andrew J. Reader, Georg Northoff

**Affiliations:** ^1^Mind, Brain Imaging and Neuroethics, University of Ottawa Institute of Mental Health ResearchOttawa, ON, Canada; ^2^Department of Biology, Carleton UniversityOttawa, ON, Canada; ^3^Department of Biology, Freie Universität BerlinBerlin, Germany; ^4^McConnell Brain Imaging Centre, Montreal Neurological Institute, McGill UniversityMontreal, QC, Canada

**Keywords:** GABA_A_ receptor, functional connectivity, eyes open, eyes closed, flumazenil, PET

## Abstract

Recent imaging studies have demonstrated that levels of resting γ-aminobutyric acid (GABA) in the visual cortex predict the degree of stimulus-induced activity in the same region. These studies have used the presentation of discrete visual stimulus; the change from closed eyes to open also represents a simple visual stimulus, however, and has been shown to induce changes in local brain activity and in functional connectivity between regions. We thus aimed to investigate the role of the GABA system, specifically GABA_A_ receptors, in the changes in brain activity between the eyes closed (EC) and eyes open (EO) state in order to provide detail at the receptor level to complement previous studies of GABA concentrations. We conducted an fMRI study involving two different modes of the change from EC to EO: an EO and EC block design, allowing the modeling of the haemodynamic response, followed by longer periods of EC and EO to allow the measuring of functional connectivity. The same subjects also underwent [^18^F]Flumazenil PET to measure GABA_A_ receptor binding potentials. It was demonstrated that the local-to-global ratio of GABA_A_ receptor binding potential in the visual cortex predicted the degree of changes in neural activity from EC to EO. This same relationship was also shown in the auditory cortex. Furthermore, the local-to-global ratio of GABA_A_ receptor binding potential in the visual cortex also predicted the change in functional connectivity between the visual and auditory cortex from EC to EO. These findings contribute to our understanding of the role of GABA_A_ receptors in stimulus-induced neural activity in local regions and in inter-regional functional connectivity.

## Introduction

Preliminary work in humans has suggested that the neurotransmitter γ-aminobutyric acid (GABA) plays a role in the brain's response to external stimuli. In the visual cortex, the magnitude of the BOLD response elicited by a visual stimulus has been shown to be negatively correlated with baseline GABA concentrations in the same region, as measured using magnetic resonance spectroscopy (MRS) (Donahue et al., 2010). This result is mirrored by the finding that regional resting GABA concentrations correlate with visual cortex gamma oscillation amplitudes (Muthukumaraswamy et al., 2009), as well as with behavioral responses to discrete visual stimuli (Edden et al., 2009). Further evidence for the involvement of GABA in stimulus induced neural activity comes from non-human primates, where BOLD responses in the visual cortex, as induced by discrete visual stimuli, have been shown to be modulated by local injections of a GABA_A_ receptor antagonist (Logothetis et al., 2010). This latter work using bicuculline in non-human primates suggests that the relationship between visual stimulus induced BOLD responses and visual cortex GABA concentrations is mediated, in part at least, through the GABA_A_ receptor. Evidence for this in humans has not been shown to date, though, leaving open the question as to what mechanisms may underlie the correlations between GABA concentrations and human visual cortex activity.

Whilst the GABA-related studies described above have focussed on the presentation of discrete visual stimuli (e.g., the switching on and off of a visual stimulus, or the alteration of the stimulus presented), the opening and closing of the eyes can also be said to represent a basic visual stimulus. Such eyes open (EO) and eyes closed (EC) conditions have been used extensively in human imaging, with these studies having shown that there are alterations in activity between the two conditions (Fox et al., 2005; Fransson, 2005; Barry et al., 2007; Yang et al., 2007; McAvoy et al., 2008; Bianciardi et al., 2009; Yan et al., 2009; Fingelkurts and Fingelkurts, 2010; Wu et al., 2010; Donahue et al., 2012).

As would be expected, these activity changes between EC and EO occur in the visual cortex, but they are also seen in other brain regions. One such region is the auditory cortex, where, in addition to changes in activity levels (Marx et al., 2003, 2004), the degree of functional connectivity between this region and the visual cortex is reduced (Wu et al., 2010). This finding of an interaction between the visual stimulus of opening the eyes and activity properties in the auditory cortex can be taken in the context of several cross-modal studies that have indicated that visual stimuli can affect the neural activity within the auditory cortex (Laurienti et al., 2002; Mozolic et al., 2008). It is not known, however, what biochemical mechanisms may underlie the activity changes in the auditory cortex itself as a result of visual stimuli. Similarly, it is not clear what transmitter systems may be involved in the changes in functional connectivity between auditory and visual cortices with the opening and closing of the eyes. With the evidence for the involvement of GABA in the visual cortex from human and animal studies described above, it would seem likely that a similar relationship between this transmitter system and visual stimulus related changes in the auditory cortex exists; however, this remains to be investigated.

Our study thus had three inter-related aims: firstly, we aimed to investigate the relationship between activity changes in the visual cortex in response to a visual stimulus (the opening of the eyes) and GABA_A_ receptors. Based on the previously shown negative correlation between GABA concentration and BOLD responses (Donahue et al., 2010), coupled with the general relationship of reduced receptor expression with increasing transmitter concentrations (Pearl et al., 2009; Deshpande et al., 2011), it was hypothesized that a positive correlation would be found between GABA_A_ receptors and BOLD responses. Secondly, we aimed to extend current knowledge of the relationship between GABA and BOLD responses from the visual cortex to the auditory cortex. As with the visual cortex, it was hypothesized that a positive correlation would be found between GABA_A_ receptors and BOLD, although this was slightly speculative as there are no analogous studies of GABA concentrations and BOLD responses in the auditory cortex to those in the visual cortex. Finally, we sought to investigate the relationship between auditory and visual cortex functional connectivity and the GABA system. As there is no relevant prior work in this area, this last line of investigation was exploratory.

**Figure A1:**
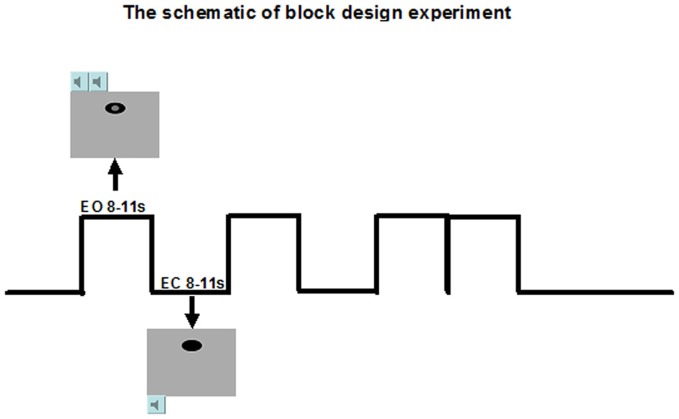
**Schemata of block-design experiment.** The first block-design experiment included eyes open and eyes closed blocks with duration from 8 to 11 s. The EC and EO blocks were pseudo-randomly presented. Ahead of each EC block, a single tone was presented to the participants; Ahead of the EO block, double tones were presented to the participants. During the EC block, an eyes closed icon was presented on the screen in front of the participants' eyes; during the EO block, an eyes open icon was presented on the screen. Should the participants made mistakes, these two icons could remind the participants what the current condition was. The participants were instructed to remain relaxed and to not fall asleep.

To these ends, we conducted an fMRI study that targeted the neural activity change from EC to EO in two different ways. In the first component a standard block-design paradigm was employed, with subjects opening and closing their eyes, that allowed the modeling of the BOLD responses induced by the EO/EC change (see Figure A1). This was followed by the scanning of longer periods of EO and EC to allow the investigation of functional connectivity between the regions of interest (ROI). As described above, studies relating BOLD responses in the visual cortex to the GABA system have involved measuring GABA concentrations using MRS (Muthukumaraswamy et al., 2009; Donahue et al., 2010). In order to investigate at the receptor level in humans it is necessary to use positron emission tomography (PET) and a suitable radio-ligand. For the GABA_A_ receptor, the ligand generally used is flumazenil (FMZ), a benzodiazepine site antagonist, labeled with ^18^F or ^11^C (Odano et al., 2009). As such, GABA_A_ receptors were imaged in the same participants using [^18^F]FMZ PET, and receptor binding potentials (BP_ND_), which represent a function of receptor density and receptor affinity, calculated (Heiss and Herholz, 2006; Innis et al., 2007).

## Materials and methods

### Participants

Twenty-seven participants took part in the study (10 Female, mean age: 22.3 years, range = from 18 to 34 years). Six of these participants had too much head motion during the fMRI scan (>3 mm) and two participants did not attend the PET scan, leaving 19 participants (8 Female, mean age: 23.1 years, range = from 18 to 34 years) with usable data. Informed written consent was obtained from all participants. The study was approved by the ethics committee of McGill University, Montreal.

### Experiment design

The first component of the fMRI experiment was a block-design paradigm consisting of EO and EC blocks with durations from 8 to11 s, pseudo-randomly presented. There were 28 blocks with EC and 28 blocks with EO. A single tone was played to indicate an EC block and a double tone to indicate an EO block. The single tone (1000 Hz) was presented at 75 dB with duration of 100 ms (10 ms up and 10 ms down). The double tone was made up of the two of these single tones, with an interval of 80 ms between them. During EC and EO blocks, icons were presented on the screen in front of the participants' eyes to remind the participants what the correct condition was, should they make any mistakes (Figure A1). The second component of the fMRI experiment was a series of longer resting-state blocks (four 119.5 s blocks, two EOs and two ECs), alternately presented. All 19 participants completed the two components of fMRI experiment. In order to evaluate whether the participants followed instructions correctly, a simple camera setup was used to monitor the participants' eyes. The participants also underwent an ^18^F-Flumazenil PET scan during which there was no task. Participants were instructed to relax and to remain awake during both the fMRI and PET scans.

### fMRI data acquisition

MR images were acquired using a Siemens 3T scanner (Siemens Trio, Erlangen, Germany), using a 32 channel headcoil. Functional images were acquired using a T2^*^-weighted echo-planar imaging (EPI) sequence (TR/TE/θ = 2270 ms/25 ms/90°, FOV = 205 × 205 mm^2^, matrix = 64 × 64 mm^2^, slice thickness = 3.2 mm, gap = 0 mm). Each volume had 47 axial slices, covering the whole brain. A high resolution T1-weighted anatomical image was also acquired.

### fMRI data processing

Functional images were processed using the AFNI software package (Cox, 1996). All fMRI data underwent a pre-processing procedure that included two- and three-dimensional head-motion corrections, masking for the removal of the skull, and spatial smoothing using a 6 mm full-width at half-maximum kernel, followed by conversion to MNI space (2 × 2 × 2 mm^3^ resolution). FWE corrections for the whole brain analysis were calculated with AlphaSim in AFNI [*p* < 0.005 uncorrected voxel-level; *p* < 0.05 FWE correction with cluster volume >61 voxels (488 mm^3^)]. In order calculate this threshold, we used all participants' gray matter masks and ran AlphaSim on each. The resulting volume thresholds ranged from 56 to 61 voxels. We selected 61 voxels as the cluster volume threshold for the study (i.e., the most conservative threshold from those calculated). To obtain the gray matter masks, the T1-weighted anatomical image was segmented into gray matter, white matter, and cerebrospinal fluid using the FAST tool from the FSL software package (http://www.fmrib.ox.ac.uk/fsl/).

#### Block-design paradigm

Following the above pre-processing procedures, the block-design fMRI data were submitted to a deconvolution analysis, based on the general lineal model (3dDeconvolve, AFNI), to obtain a map of the estimated coefficients (β value) for the contrast [EO > EC]. A one-sample *t*-test (*n* = 19) was then carried out on the estimated coefficients maps of the contrast [EO > EC] at the group level. Based on the group results, we found activated clusters in seven cortical regions: the visual cortex, auditory cortex, posterior cingulate cortex (PCC), right temporoparietal junction (rTPJ), left temporoparietal junction (lTPJ), caudal cingulate cortex (cACC), and perigenual anterior cingulate cortex (pACC). For these seven regions, we created ROIs based on the most significantly activated voxels in each. This approach was used rather than a sphere centered on the peak voxel in order to retain information about activation distribution. The significant voxel cluster size threshold used was 64 voxels, corresponding to the volume of activation cluster in the pACC at a significance threshold of *p* < 0.05, FWE corrected, the smallest of the clusters obtained (see Table A1 for more detailed information). Note that in the PCC two separate activity clusters were found—the larger and more significant of these was used for the PCC ROI.

**Figure A2:**
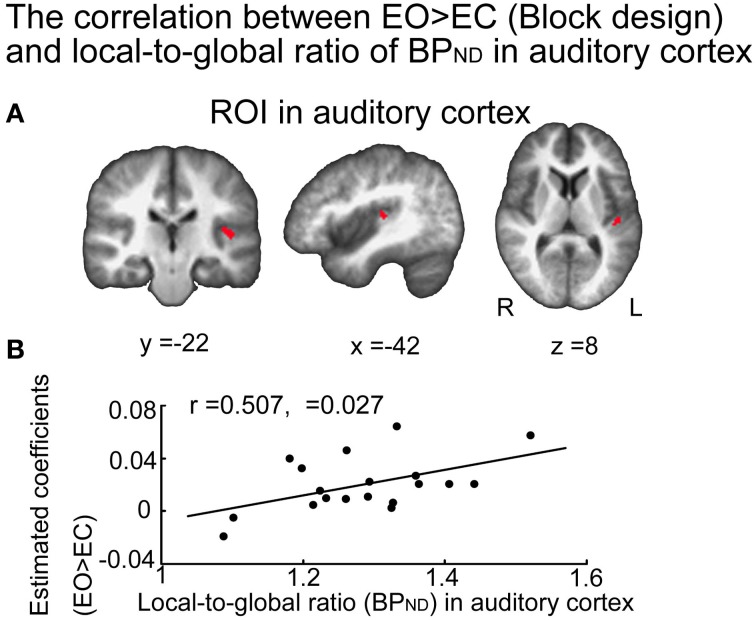
**The correlation between the estimated coefficients (EO > EC) and the local-to-global ratio of GABA_A_ receptor BP_ND_ (white matter as reference region) in the left auditory cortex. (A)** Activated regions in the left auditory cortex that are taken as the ROI. **(B)** Positive correlation between the estimated coefficient of the contrast EO > EC and the local-to-global ratio of GABA_A_ receptor BP_ND_ in the left auditory cortex (*n* = 19) (*r* = 0.507, *p* = 0.027 uncorrected).

The seven cortical ROIs were then applied to the map of estimated coefficients for the contrast [EO > EC], and the mean β values within them calculated for each subject. The visual (see Figure 3A for location) and auditory cortex (see Figure A2A for location) ROIs were the primary target of the study. The bilateral TPJ, cACC, pACC, and PCC ROIs were used, firstly, as control regions for results in the visual and auditory ROIs. In addition, as prior studies have shown neural activity changes in the so-called “default-mode” network between EO and EC (Yan et al., 2009), the bilateral TPJ, cACC, pACC, and PCC ROIs were used for supplementary investigation of the relationship between GABA_A_ receptor BP_ND_ and the neural activity in these regions.

#### Longer resting state paradigm

Head motion and other noise effects (signal from the white matter and CSF) were removed from the functional data through linear regression. The visual cortex ROI, as the functional connectivity seed region, was applied to calculate the mean time series of the resting state data in this region during EC and EO. The mean time series from the visual cortex were then used to calculate the functional connectivity between the visual cortex and the all voxels in the brain during EC and EO through Pearson correlation. Fisher's Z transformation was used to transform the *R* value of the correlation to a normally distributed *Z*-value. The ROIs from the block-design experiment were applied to calculate mean functional connectivity *Z*-values during EC and EO for each subject. These were then compared using a paired *t*-test (*n* = 19) to investigate the change of the functional connectivity from EC to EO.

In order to investigate the variability of the resting state during EC and EO, we calculated the reciprocal of the standard deviation (SD) of the time series of resting state during EC and EO in each voxel. The ROIs from the block-design experiment were then applied to draw the mean value of the reciprocal of SD in each ROI during EC and EO. These were then entered into a paired *t-test* (*n* = 19) to investigate the change in variability between EC and EO.

Results of the comparisons between EO and EC resting-state functional connectivity and SD are reported at both an uncorrected threshold of *p* < 0.05 and at a threshold corrected for multiple comparisons according to the Bonferroni approach. There were a total of six comparisons of function connectivity between EC and EO (i.e., functional connectivity between the visual cortex and each of six other regions), giving a threshold of *p* < 0.008 for Bonferroni correction in this analysis. In the SD analysis, seven regions were included, giving a Bonferroni corrected threshold of *p* < 0.007.

#### PET data acquisition and processing

Nineteen subjects underwent positron-emission-tomography (PET) imaging with FMZ, a benzodiazepine antagonist that binds at the GABA_A_ benzodiazepine site. This method has been widely used to measure GABA_A_ receptor density *in vivo* in humans (Frey et al., 1991; Salmi et al., 2008). PET imaging was done randomly either before or after the fMRI scan (mean duration ± SD between both types of scans = 1.9 ± 3.6 days).

Whole-brain [^18^F] FMZ BP_ND_ values were obtained using a Siemens ECAT HRRT (High Resolution Research Tomograph) PET system (Siemens Medical Solutions, Knoxville, TN, USA) (de Jong et al., 2007). [^18^F]FMZ was synthesized as published previously (Massaweh et al., 2009). Head movement was minimized with a head-restraining adhesive band. A six-minute transmission scan (^137^Cs-point source) was first acquired for attenuation correction followed by an intravenous tracer injection (over 60 s) of 260.7 MBq (± 21.24 SD) of [^18^F]FMZ. Subjects were instructed to close their eyes and remain awake.

**Figure 1:**
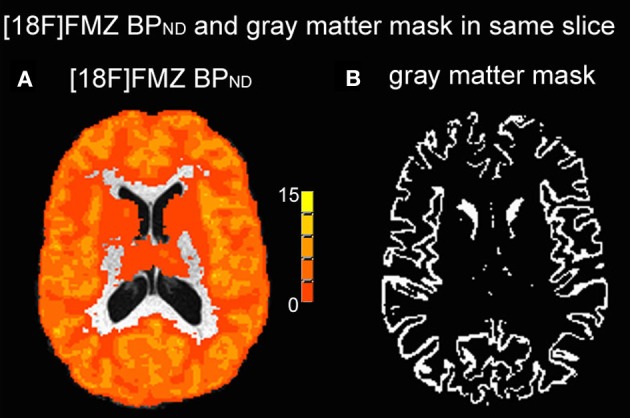
**(A)** Example [^18^F] FMZ BP_ND_ image without partial volume correction (white matter as the reference region). **(B)** Example gray matter mask with a gray matter proportion threshold of 0.9 in the same slice as **(A)**.

List-mode data were acquired for a period of 60 min and then binned into a series of 26 sequential sets of 2209 span 9 sinograms of increasing temporal duration, ranging from 30 s to 5 min. PET data were reconstructed using a 3D OP-OSEM algorithm (10 iterations and 16 subsets) (Hudson and Larkin, 1994; Hong et al., 2007) with full accounting for scatter, random coincidences, attenuation, decay, dead-time, and frame-based motion correction (Costes et al., 2009). The images used had a voxel size of 1.22 × 1.22 × 1.22 mm^3^ (256 × 256 × 207 voxels). GABA_A_ BP_ND_ maps were then calculated according to the Logan plot method, using the bilateral cerebral white matter as the reference tissue region (Logan et al., 1996; la Fougere et al., 2011). Reference region centers were defined in MNI space on the standard MNI template (center coordinates = 28, −14, 32 and −26, −12, 34). The central points were then converted into individual PET space through the previously calculated MNI to PET transformations. Five millimeter radius spheres were then made around these central points and these used as reference regions. The mean value from the two regions was used in the Logan analysis. BP_ND_ maps were linearly aligned to each subject's anatomical images using the AFNI toolbox (Figure 1A).

As described above, T1-weighted anatomical images were segmented into gray, white, and CSF compartments using the FSL tool, FAST. The gray matter proportion maps were used in a number of steps intended to reduce partial volume effects. This involved firstly thresholding the gray matter proportion maps at 0.9 (in a range from 0 to 1) to create a mask of high-probability gray matter voxels (Figure 1B) and then applying this to the BP_ND_ maps. Since the GABA_A_ receptor is predominantly located in gray matter, rather than white (Heiss and Herholz, 2006), in order to focus on the BP_ND_ in gray matter we used a mask with this high gray matter proportion. Secondly, GABA_A_ receptor BP_ND_ maps were then corrected for partial volume effects on a voxel-wise basis by dividing by the gray matter proportion within each voxel following spatial smoothing using a 2.5 mm at FWHM kernel (corresponding to the PET scanner resolution) (Giovacchini et al., 2005; la Fougere et al., 2011). All BP_ND_ values for subsequent analyses were taken from within the masked gray matter region. Finally, the T1-weighted anatomical images from each participant were linearly transformed into MNI space and these transformation matrices combined with the PET-to-anatomical ones to give PET-to-MNI transforms. Using the inverse of these, the ROIs from the block-design experiment in MNI space were transformed into individual PET space.

Two approaches to the GABA_A_ receptor BP_ND_ values were adopted in the analysis. The first of these was to use the absolute BP_ND_ values obtained for each region for correlation with activity changes and functional connectivity. The second approach took local BP_ND_ values as a ratio to the average BP_ND_ value across the whole cortex for the same correlations. The rationale for using this second value in addition to the absolute BP_ND_ was that the general BP_ND_ level in the cortex may be variable across the participants. The same BP_ND_ in one brain region may thus reflect a different relative level in different people. If there is variance in general GABA_A_ receptor function between people such that the same BP_ND_ was linked to somewhat different physiological responses, then the relevant variance would be lost when correlating with absolute values. In current study, the BP_ND_ in each of the seven ROIs (visual cortex, auditory cortex, pACC, cACC, PCC, and bilateral TPJ) was significantly correlated with BP_ND_ in the whole brain (*p* ≤ 0.001). The use of the local-to-global ratio is similar to PET studies of regional blood flow and oxygen consumption studies (Raichle et al., 2001).

Pearson correlation analyses between the two BP_ND_ measures (absolute and local-to-global ratio) and estimated coefficients (EO > EC) and functional connectivity *Z*-values in the seven ROIs were calculated (SPSS Inc., Chicago, IL). The significance level for correlations was *p* < 0.05, uncorrected, and *p* < 0.007 with Bonferroni correction. For all the datasets, the normality of datasets was tested using the Shapiro–Wilk test. In order to extend the ROI-based correlation results, we also used the local-to-global ratio of GABA_A_ receptor BP_ND_ in the visual cortex to correlate with the difference of functional connectivity (EC-EO) (visual cortex as seed region) at the voxel level across the whole-brain. The difference in functional connectivity was calculated as the *Z*-value of FC during EC minus the *Z*-value of FC during EO.

As there are some questions regarding the optimal reference region to use for the Logan method (Lammertsma and Hume, 1996; Klumpers et al., 2008; Frankle et al., 2009), correlations between BP_ND_ and estimated [EO > EC] coefficients and functional connectivity *Z*-values were repeated using BP_ND_ values calculated using the pons as the reference region. Similar results were obtained as with the original white matter reference region BP_ND_ (see “Appendix” for details).

## Results

**Figure 2:**
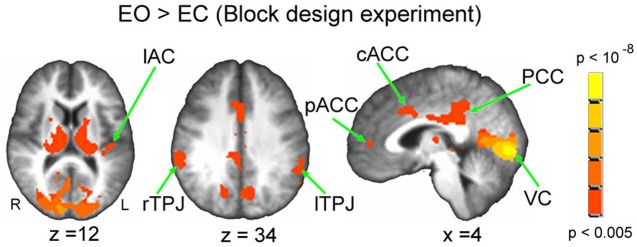
**Activated brain areas based on the contrast EO > EC from the block-design experiment.** The results were significant at *p* < 0.05 (FWE corrected). For display purposes, the results are exhibited with a threshold of *p* < 0.005 uncorrected. PCC, posterior cingulate cortex; rTPJ, right temporoparietal junction; lTPJ, left temporoparietal junction; cACC, caudal cingulate cortex; pACC, perigenual anterior cingulate cortex; VC, visual cortex; lAC, left auditory cortex.

Based on the block-design experiment, compared with EC, EO showed stronger signal changes in the visual cortex (VC), left auditory cortex (lAC), PCC, rTPJ, lTPJ, cACC, and pACC (Figure 2, Table 1). Signal changes were also seen in the thalamus—this region was not investigated further, however, as the focus was on the cortex. No regions showed stronger activity during EC than during EO. The ROIs created from the cortical clusters of activation (see section “Materials and Methods” for details) were used to extract the estimated coefficients for the [EO > EC] contrast, along with the GABA_A_ receptor BP_ND_ for these regions. The mean BP_ND_ in each of the ROIs was divided by the global mean BP_ND_ to obtain BP_ND_ local-to-global ratios.

**Table 1:** **Activated clusters during EO > EC (Block design experiment)**.

**Brain regions**	**Coordinates (MNI)**			***T*-value**	**Volume (mm^3^)**
	***x***	***y***	***z***		
Visual cortex	10	−84	0	8.81	52408
Right thalamus	28	−29	3	12.00	13616
Left thalamus	−22	−26	−9	12.40	12784
PCC	2	−38	28	8.32	6432
rTPJ	62	−34	39	5.23	2280
lTPJ	−54	−47	36	4.83	2040
cACC	8	18	40	5.18	1672
PCC	−8	−75	33	6.82	1536
Left auditory cortex	−44	−23	10	5.03	912
pACC	6	49	5	4.48	512

*Note: The results were threshold at p < 0.05 (FWE corrected). The T-value given represents the highest value in each cluster. PCC, posterior cingulate cortex; rTPJ, right temporoparietal junction; lTPJ, left temporoparietal junction; cACC, caudal cingulate cortex; pACC, perigenual anterior cingulate cortex*.

**Figure 3:**
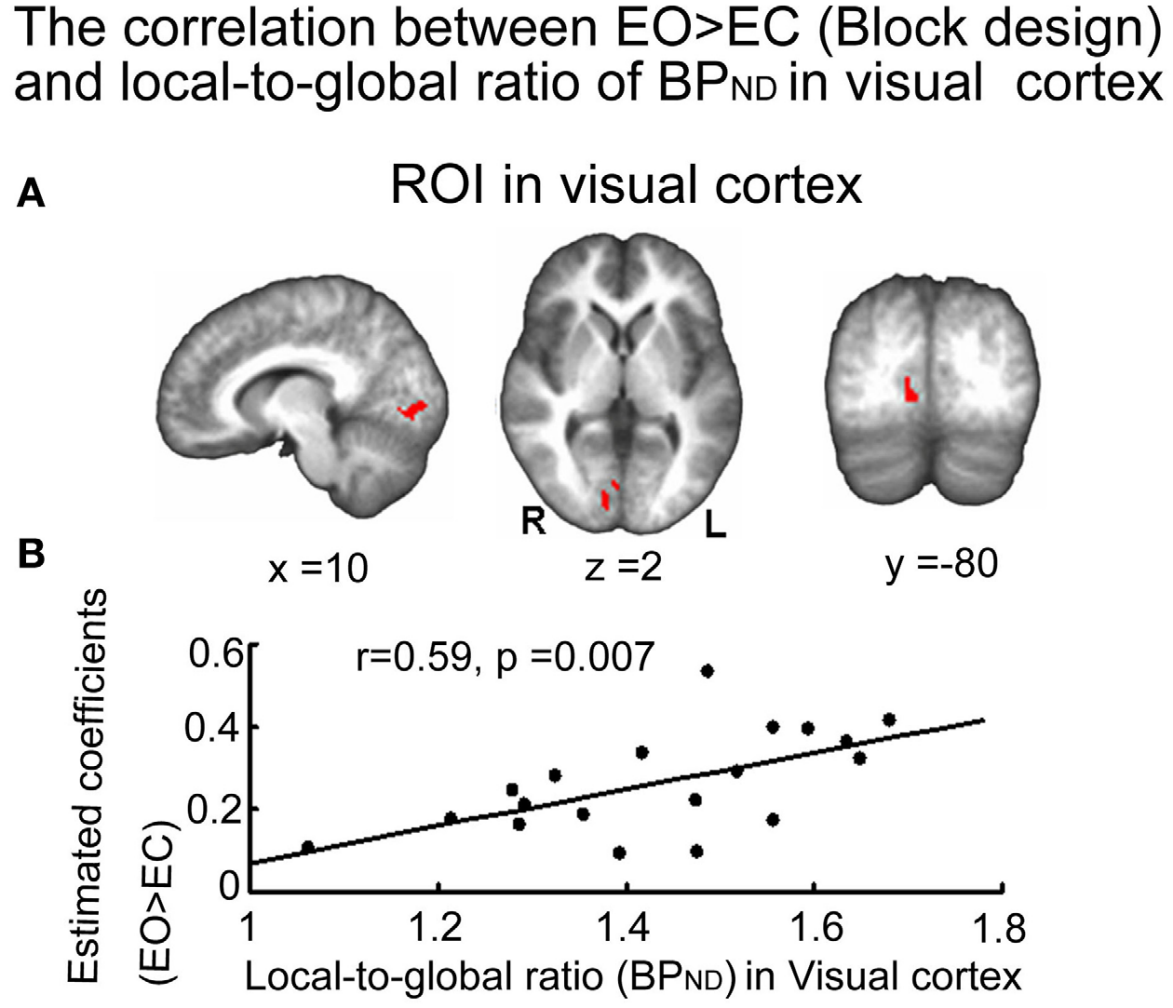
**Correlation between estimated coefficients (EO > EC) and the local-to-global ratio of GABA_A_ receptor BP_ND_ in the visual cortex. (A)** Activated regions in the visual cortex during eyes open (EO) when compared to eyes closed (EC) taken as ROIs. **(B)** Positive correlation between the estimated coefficients for the contrast [EO > EC] and the local-to-global ratio of GABA_A_ receptor BP_ND_ in the visual cortex [*r* = 0.59, *p* = 0.007 uncorrected (*p* < 0.05, with Bonferroni correction)] (*n* = 19).

The estimated coefficients for EO > EC in visual cortex were positively correlated with the local-to-global ratio of GABA_A_ receptor BP_ND_ in the same region [*r* = 0.599, *p* = 0.007 uncorrected (*p* < 0.05, with Bonferroni correction)] (Figure 3B): the higher the GABA_A_ receptor binding potential relative to global BP_ND_, the higher was the degree of BOLD signal change during EO > EC. The absolute GABA_A_ receptor BP_ND_ did not show a significant correlation with the estimated coefficient of EO > EC.

**Figure A3:**
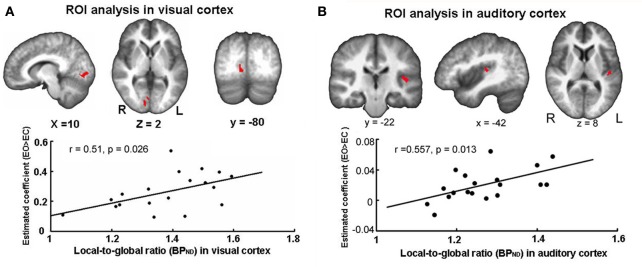
**Correlation between the estimated coefficients (EO > EC) and the local-to-global ratio of GABA_A_ receptor BP_ND_ (calculated with pons as reference region) in visual and auditory cortex. (A)** Upper panel shows activated regions in the visual cortex during eyes open (EO) when compared to eyes closed (EC) that are taken as ROIs. Lower panel shows a positive correlation between the estimated coefficients of the contrast EO > EC and the local-to-global ratio of GABA_A_ receptor BP_ND_ in the visual cortex (*n* = 19). **(B)** Upper panel shows activated regions in the left auditory cortex that are taken as ROIs. Lower panel shows a positive correlation between the estimated coefficient of the contrast EO > EC and the local-to-global ratio of GABA_A_ receptor BP_ND_ in the auditory cortex (*n* = 19).

Analogous results to the visual cortex were obtained in the auditory cortex, where the estimated coefficient of EO > EC were positively correlated with the local-to-global ratio of GABA_A_ receptor BP_ND_ (*r* = 0.507, *p* = 0.027 uncorrected) (Figure A2B), but not the absolute GABA_A_ receptor BP_ND_ values. Similar results for both the visual and auditory cortices were also seen when using of the BP_ND_ values calculated using the pons as the reference region (Figure A3). There was no correlation between the estimated coefficient of EO > EC and the local-to-global ratio of GABA_A_ receptor BP_ND_ or absolute GABA_A_ receptor BP_ND_ within PCC, lTPJ, rTPJ, cACC, and pACC, respectively.

**Figure 4:**
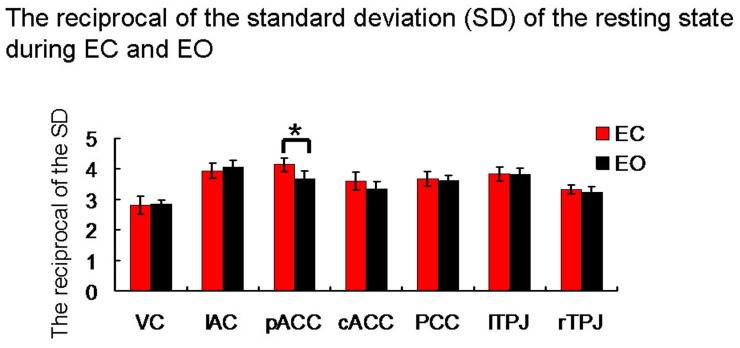
**The reciprocal of the standard deviation (SD) of the resting state during both EC and EO.** VC, visual cortex; lAC, left auditory cortex; PCC, posterior cingulate cortex; rTPJ, right temporoparietal junction; lTPJ, left temporoparietal junction; cACC, caudal cingulate cortex; pACC, perigenual anterior cingulate cortex. ^*^means there is a significant difference (*p* < 0.05 uncorrected).

Resting state time course variability did not change from EC to EO in any of the visual cortex, auditory cortex, PCC, bilateral TPJ, or cACC. However, in the pACC, the reciprocal of the SD of the resting state during EC was higher than during EO (*t* = 2.69, *p* = 0.015). In the other words, the variability of the resting state activity in the pACC during EO was significant higher than during EC (Figure 4), but was the same in all other ROIs.

**Figure 5:**
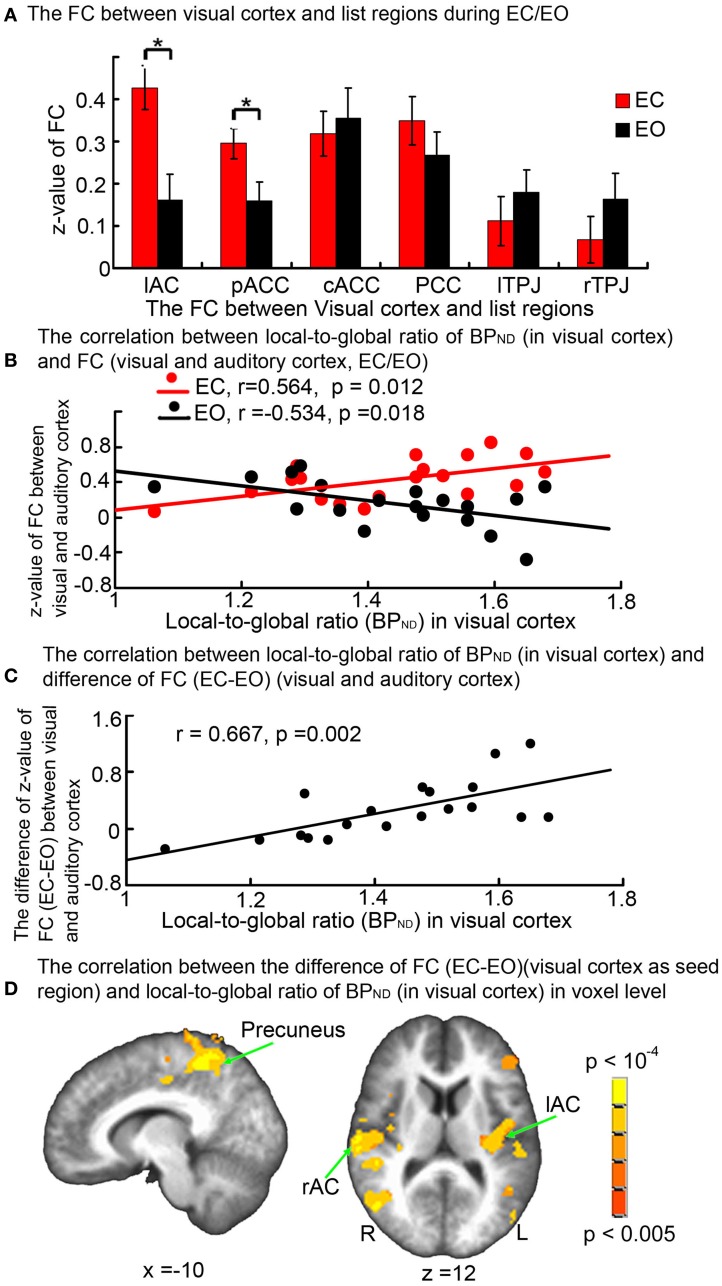
**(A)** Histogram of *Z*-values for the functional connectivity between visual cortex and activated regions from contrast [EO > EC] (block design experiment) during EC and EO. FC, Functional connectivity. ^*^indicates *p* < 0.05 uncorrected. Compared with EC, FC between the visual and auditory cortices was significantly reduced during EO (*t* = 2.87, *p* = 0.01 uncorrected), as was FC between visual cortex and pACC (*t* = 2.46, *p* =0.024 uncorrected). **(B)** FC between the visual and auditory cortices during EC was positively correlated with the local-to-global ratio of GABA_A_ receptor BP_ND_ in visual cortex (*r* = 0.564, *p* = 0.012 uncorrected); in contrast, during EO, the relationship becomes negative (*r* = −0.534, *p* = 0.018 uncorrected). **(C)** Difference (EC-EO) in FC between the visual and the auditory cortex was significantly correlated with local-to-global ratio of GABA_A_ receptor BP_ND_ in the visual cortex [*r* = 0.667, *p* = 0.002 (*p* < 0.05, with Bonferroni correction)]. **(D)** Voxel-wise regression of local-to-global ratio of GABA_A_ receptor BP_ND_ in the visual cortex ROI against whole-brain FC *Z*-value difference (EC-EO) (visual cortex ROI as seed region); significant clusters in bilateral auditory cortex are shown (*p* < 0.05, FWE corrected).

Compared with EC, the functional connectivity between the visual and auditory cortices was significantly reduced during EO (*t* = 2.87, *p* = 0.01 uncorrected). This was also the case with the functional connectivity between the visual cortex and the pACC (*t* = 2.46, *p* = 0.024 uncorrected). The functional connectivity between the visual cortex and the other ROIs (PCC, rTPJ, lTPJ, and cACC) did not change between EC and EO (Figure 5A).

Functional connectivity between the visual and auditory cortices during EC was positively correlated with the local-to-global ratio of GABA_A_ receptor BP_ND_ in the visual cortex (*r* = 0.564, *p* = 0.012 uncorrected). There was no significant correlation with the absolute GABA_A_ receptor BP_ND_. In contrast to EC, the functional connectivity between the visual and auditory cortex during EO was negatively correlated with the local-to-global ratio of GABA_A_ receptor BP_ND_ in visual cortex (*r* = −0.534, *p* = 0.018 uncorrected) (Figure 5B), but not the absolute GABA_A_ receptor BP_ND_ in the visual cortex. The correlation between visual-auditory functional connectivity and visual local-to-global GABA_A_ receptor BP_ND_ was not seen when the auditory cortex GABA_A_ receptor BP_ND_ values were instead used. Functional connectivity between the visual cortex and regions other than the auditory cortex (PCC, rTPJ, lTPJ, pACC, and cACC) did not show any correlation with either the local-to-global ratio of GABA_A_ receptor BP_ND_ or the absolute GABA_A_ receptor BP_ND_ in the visual cortex during EC and EO. There was no correlation between the differences in visual cortex functional connectivity to each of these regions and the local-to-global ratio of GABA_A_ receptor BP_ND_ within them.

**Figure A4:**
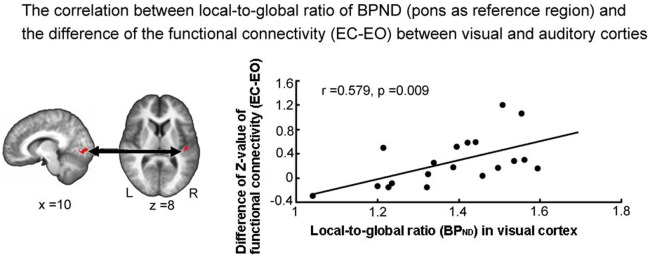
**The functional connectivity difference (EC minus EO) correlated with local-to-global ratio of BP_ND_ (pons as reference region) of GABA_A_ receptor in visual and auditory cortex (*r* = 0.579, *p* = 0.009 uncorrected).** Fisher's Z-transformation was used to translate the *R*-value of the correlation between visual and auditory cortex time series to a normally distributed *Z*-value.

Finally, the difference between functional connectivity (EC-EO) between visual and auditory cortex was positively correlated with the local-to-global ratio of GABA_A_ receptor BP_ND_ in visual cortex [*r* = 0.667, *p* = 0.002 uncorrected, (*p* < 0.05, Bonferroni correction)] (Figure 5C), was not significantly correlated with absolute GABA_A_ receptor BP_ND_. Similar results were also shown by using of the BP_ND_ calculated using the pons as reference region (Figure A4). In contrast, the difference in functional connectivity (EC-EO) between visual and auditory cortex was not correlated with the local-to-global ratio of GABA_A_ receptor BP_ND_ or the absolute GABA_A_ receptor BP_ND_ in the auditory cortex.

In order to identify any other correlating regions, we also used the local-to-global ratio of GABA_A_ receptor BP_ND_ in the visual cortex to correlate with the difference of functional connectivity (EC-EO) (visual cortex as seed region) at the voxel level across the whole brain. The voxel-level results confirmed the correlation between the local-to-global ratio of GABA_A_ receptor BP_ND_ in the visual cortex and the difference in functional connectivity (EC-EO) between the visual and auditory cortices. The results also showed a correlation between the local-to-global ratio of GABA_A_ receptor BP_ND_ in the visual cortex and the difference in functional connectivity (EC-EO) between visual cortex and a number of other regions, such as the precuneus (Figure 5D, Table A2).

## Discussion

We report for the first time in humans on the relationship between GABA_A_ receptor BP_ND_ and neural activity change between the EC and EO conditions. More specifically, our findings show that the local-to-global ratio of GABA_A_ receptor BP_ND_ in the visual cortex positively predicts the degree of change in neural activity in the same region between EC and EO. Moreover, we observed the change in the degree of functional connectivity between the visual and auditory cortices from EC to EO to be correlated with the local-to-global ratio of GABA_A_ receptor BP_ND_ in the visual cortex.

Taken together, our findings demonstrate a correlation between GABA_A_ receptor BP_ND_ local-to-global ratios and changes in intra- and inter-regional activity properties between EC and EO in the visual cortex. This would lend support to the hypothesis that GABAergically mediated activity may contribute to the brain activity induced by external stimuli in the sensory cortices.

Our results demonstrated significant changes in neural activity in both the auditory and visual cortices between the EC and EO condition. This is in accordance with previous studies showing similar activation patterns in the auditory and visual cortices during EO when compared to EC (Marx et al., 2003; Gonzalez-Castillo et al., 2012). The link between a visual stimulus and activity changes in the auditory cortex is also in accordance with prior studies demonstrating this form of multi-modal interaction (Laurienti et al., 2002; Mozolic et al., 2008). Our results extend these prior findings by demonstrating that the difference in neural activity between EC and EO in both the visual and auditory cortices is correlated with the local-to-global ratio of GABA_A_ receptor BP_ND_ in those regions: the greater the local-to-global ratio of GABA_A_ receptor BP_ND_, the higher the change in neural activity from EC to EO. This reinforces prior work relating GABA and BOLD responses in the visual cortex and provides early evidence in humans that the same processes are involved in the auditory cortex.

The correlation between local-to-global ratio of GABA_A_ receptor BP_ND_ and BOLD signal changes during EO when compared to EC was shown here to be positive. This contrasts with the negative correlations previously reported between the concentration of (intra- and extra-cellular) GABA, as measured using MRS, and stimulus-induced BOLD signal changes in the visual cortex (Muthukumaraswamy et al., 2009). GABA_A_ receptor binding potentials and GABA concentration thus predict the same measure (i.e., the BOLD signal) in opposite ways. This mirrored relationship is in line with a situation whereby increases in transmitter release or availability lead to compensatory decreases in receptor numbers to maintain a receptor-transmitter balance. Whilst to our knowledge no studies have directly demonstrated such a relationship in humans, some studies in animals (Deshpande et al., 2011) and humans (Pearl et al., 2009) strongly suggest that this is indeed the case and that it is likely related to the activation of molecular cascades associated with GABA_A_ receptor trafficking (Arancibia-Carcamo and Kittler, 2009). In this context, it is also worth noting that activation of presynaptic GABA_A_ autoreceptors is well-known to inhibit the release of GABA into the synapse (Axmacher and Draguhn, 2004); this may be one way in which negative feedback is used to dynamically regulate the receptor-transmitter balance. Where we measure reduced GABA_A_ receptor values, then, one could infer that this reflects an increased release of GABA transmitter. With this knowledge, we can more strongly infer that the results obtained in previous MRS studies (Donahue et al., 2010) reflect a correlation between the stimulus-induced BOLD responses and GABA that is actually released and is acting at a receptor (rather than between the BOLD and some other unknown process that is reflected in the concentration measures). Finally, our results demonstrate that the GABA_A_ receptor subtype is relevant to the MRS GABA concentration and BOLD response correlations seen, and thus to the relationship between the GABAergic system and activity changes in response to visual stimuli in the visual and auditory cortex (whilst not ruling out that other GABA receptor subtypes may also play a role).

These results show that the local-to-global ratio of GABA_A_ receptor BP_ND_ in the visual cortex was significantly correlated with estimated coefficients from the [EO > EC]. In contrast, the absolute GABA_A_ receptor BP_ND_ values were not significantly correlated with the coefficients (although some comparisons had a similarly positive *r*-value and *p*-values close to 0.1, which may be suggestive of a relationship requiring additional investigation with a larger sample). This is in contrast to work by Wiebking et al. (2012) in which they found correlations between absolute GABA_A_ receptor BP_ND_ values and BOLD responses in anterior and posterior midline regions during a task. This difference may be due to the slightly different values being correlated in that work to this, where they focussed on the difference between two separate percentage signal change values, rather than the single condition parameter estimates used here. Indeed, where Wiebking et al. did correlate single condition values with GABA_A_ receptor BP_ND_ values, there was no significant correlation, although interestingly there were similar correlation coefficients as seen in the present work. Alternatively, the difference between this prior study's results and those obtained here may reflect a situation in which the regional physiological effects seen are determined by the relationship between GABA_A_ receptors in the target region and the levels of the same across other regions of the brain (reflected by the local-to-global ratio). Thus, the patterns of activity seen would not be entirely the product of isolated local GABA_A_ receptor properties (assumedly representing local inhibition), but would result from interactions between local and global properties. This latter proposal has compelling aspects when considering the brain as a network of interacting regions, but requires future investigation in order to characterize it fully.

In addition to local signal changes in the auditory and visual cortices, we also investigated the functional connectivity between these regions, observing a decrease in the degree of functional connectivity during EO when compared to EC. This is consistent with previous work showing that the functional connectivity between the right lingual gyrus and superior temporal gyrus decreased from EC to EO (Wu et al., 2010). Most importantly, our results demonstrate that the decrease in auditory-visual functional connectivity from EC to EO could be predicted by the local-to-global ratio of GABA_A_ receptor BP_ND_ in the visual cortex (but not GABA_A_ receptor BP_ND_ local-to-global ratio in the auditory cortex): the higher the local-to-global ratio of GABA_A_ receptor BP_ND_ within the visual cortex, the more the strength of the functional connectivity between the visual and auditory cortices decreases from EC to EO. It should be noted, however, that this relationship between the local-to-global ratio of GABA_A_ receptor BP_ND_ and functional connectivity changes from EC to EO may reflect the opposing correlations between local-to-global ratio of GABA_A_ receptor BP_ND_ and functional connectivity during EC and during EO (i.e., the subtraction of decreasing values in EO from increasing ones in EC). Given the observed correlation, one may consequently suggest that the changes in local neural activity and functional connectivity from EC to EO can be traced back, in part, to the degree of GABA_A_ receptor binding potential and thus to GABA release. Furthermore, this may provide a cue for investigations of the mechanism of cross-modal effect between visual and auditory cortices (Laurienti et al., 2002; Mozolic et al., 2008). Additionally, voxel-level results also showed a correlation between the local-to-global ratio of GABA_A_ receptor BP_ND_ in the visual cortex and the difference in functional connectivity (EC-EO) between the visual cortex and other regions, most particularly the precuneus. The finding of a relationship with the precuneus is suggestive in the context of recent findings that posterior medial GABA concentrations are negatively correlated with resting-state functional connectivity in the so-called default mode network (Kapogiannis et al., 2012), pointing to there being a brain-wide relationship between resting-state properties and the GABAergic system.

Finally, some limitations of the present study need to be taken into account. Firstly, we neither quantified the visual input subjects received (or perceived) when opening their eyes, nor did we include any behavioral measures relating to stimulus-induced activity; this though is necessary to further investigate the behavioral and psychological relevance of GABA. Secondly, one may also include a more subtle measure of EO, such as EO in darkness and brightness, which may further typify the difference between EC and EO suggested here. Thirdly, not all results were significant at the relevant Bonferroni thresholds, although the major findings of a correlation between visual cortex GABA_A_ receptor BP_ND_ local-to-global ratio and both EO >EC signal changes and the EO to EC change in the degree of functional connectivity between the visual and auditory cortices were significant at this level. The uncorrected results must therefore be interpreted with caution and may be taken as starting points for future, confirmatory, studies.

In summary, it was shown that measures of GABA_A_ receptors are correlated with activity changes in the visual cortex in response to the opening of the eyes. This supports and advances prior work involving MRS measures of resting GABA in the same region by confirming that GABA release is involved in the correlation between GABA concentration and BOLD responses and by identifying the involvement of the GABA_A_ receptor. It was then shown that the same relationship between GABA_A_ receptors and activity changes in response to the opening of the eyes was present in the auditory cortex, providing an early link between GABA and BOLD responses in this area in humans. Finally, it was shown that GABA_A_ receptors are implicated in the strength of functional connectivity between the visual and auditory cortices, and between the visual cortex and non-sensory regions.

### Conflict of interest statement

The authors declare that the research was conducted in the absence of any commercial or financial relationships that could be construed as a potential conflict of interest.

## Appendix

**Table A1:** **ROIs defined based on the EO > EC (block-design experiment)**.

**ROIs from block-design experiment**	**Coordinates (MNI)**			**Volume (mm^3^)**
	***x***	***y***	***z***	
Visual cortex	10	−82	−5	66
Auditory cortex	−39	−26	12	67
cACC	2	11	40	67
lTPJ	−54	−49	40	64
rTPJ	62	−37	39	67
pACC	3	47	6	64
PCC	1	−44	44	65

*Note: The coordinates represent the center of each ROI. PCC, posterior cingulate cortex; rTPJ, right temporoparietal junction; lTPJ, left temporoparietal junction; cACC, caudal cingulate cortex; pACC, perigenual anterior cingulate cortex*.

**Table A2:** **Activated clusters involved in the correlation between local-to-global ratio BP_ND_ (white matter as reference region) (in visual cortex) and the difference of FC (EC-EO) (visual cortex as seed region)**.

**Brain regions**	**Coordinates (MNI)**			***F*-value**	**Volume (mm^3^)**
	***x***	***y***	***z***		
Right auditory cortex	62	−10	2	39.34	10936
Precuneus	10	−48	60	30.54	8264
Left auditory cortex	−44	−21	25	36.75	6112
Right middle temporal gyrus	52	−53	−15	42.82	4720
Left middle temporal gyrus	−40	−71	22	33.94	2368
Left inferior frontal gyrus	−48	38	15	33.06	1664
Right postcentral gyrus	56	−21	55	24.88	1624
Supplementary motor area	−10	−23	59	27.35	1272

*Note: *p* < 0.05 (FWE corrected). F-value is the peak value of each cluster*.
